# Cutaneous Adverse Reactions of Immunotherapy in Patients with Advanced Melanoma

**DOI:** 10.3390/cancers15072084

**Published:** 2023-03-31

**Authors:** Vasiliki Nikolaou, Antonis Tsimpidakis, Alexander Stratigos

**Affiliations:** 1st Dermatology Department, “Andreas Syggros” Hospital for Skin Diseases, National & Kapodistrian University of Athens, 16121 Athens, Greece; tsimpidakis.antonis@gmail.com (A.T.);

**Keywords:** cutaneous irAEs, melanoma, immunotherapy, immune checkpoint blockers

## Abstract

**Simple Summary:**

It is estimated that 30–50 percent of patients treated with Immune Checkpoint Blockers will eventually develop cutaneous immune-related adverse events. These toxicities are in, most of the time, low-grade reactions; however, they are characterized by a wide clinical spectrum. Clinicians who utilize these novel agents must have a thorough understanding of the pathogenesis, clinical characteristics, as well as the proper treatment of these toxicities. In this review, we analyze the treatment approaches as well as unique features observed in melanoma patients who develop cutaneous immune-related adverse events.

**Abstract:**

Immune checkpoint blockers (ICBs) have been widely used during the last decade for the treatment of various tumors, including advanced and metastatic melanoma. While these agents have improved melanoma patients’ survival rates, they have also been associated with various autoimmune toxicities, with the skin being most commonly affected. The severity of cutaneous toxicity can not only negatively affect patients’ quality of life but can also limit the proper treatment of cancer. Thus, the role of the dermatologist is substantial in early detecting and promptly treating these adverse events. Maculopapular rash, psoriasiform, lichenoid dermatoses and bullous pemphigoid are the most frequent cutaneous adverse events that require immediate intervention. Other rare autoimmune toxicities, e.g., sarcoidosis, dermatomyositis or subacute lupus, have also been reported. In this review, we summarize the aspects of ICB-induced cutaneous toxicities in patients with melanoma, emphasizing their management and treatment options in clinical practice.

## 1. Introduction

Immune checkpoint blockers (ICBs) have revolutionized the treatment of melanoma, improving long-term survival rates for patients with advanced disease [1,2]. During the past decade, the relative survival rate for patients with advanced-stage melanoma has increased from 20.6–39.3% [3]. The 5-year overall survival of the patients being treated with the combination of ipilimumab and nivolumab for metastatic melanoma is 50% [1]. Given that ICBs have been approved for the treatment of patients with solitary skin disease in an adjuvant setting, the number of patients exposed to these agents has steadily increased. These agents target immune-cell-surface checkpoint proteins, including the cytotoxic T-lymphocyte-associated protein-4 (CTLA4) and the programmed death-1/programmed death ligand-1 (PD-1/PD-L1), which inhibit anti-cancer immune responses. Recently another treatment combination targeting Lymphocyte-activation gene 3 (LAG-3) and PD-1 has been approved for the treatment of metastatic melanoma. LAG-3 is a cell-surface molecule that is expressed on immune cells, including T cells, and negatively regulates T-cell proliferation and effector T-cell function [4]. ICBs activate the immune system in a non-specific manner; therefore, it is not surprising that they can induce toxicities that mimic autoimmune disorders. The most commonly affected organ is the skin. In clinical practice, cutaneous immune-related adverse events (irAEs) can present in various forms and mimic most inflammatory and autoimmune skin disorders. It should be noted that although these toxicities tend to be low grade, they can be dose-limiting and negatively impact patient quality of life. The purpose of this paper is to review cutaneous irAEs and discuss their unique features observed in patients with melanoma.

## 2. Cutaneous irAEs in Melanoma Patients

Cutaneous irAEs are the first and most frequent toxicities to occur with the use of ICBs. Although great efforts have been made to differentiate the clinical types of skin toxicities, their exact frequency and prognostic significance remain uncertain. Morbilliform rashes, eczematous reactions, psoriasis, vitiligo, bullous diseases, and lichenoid reactions are among the most common toxicities. Dual inhibition of CTLA-1 and PD-1 leads to a higher incidence (58.5–71.5%) rate of cutaneous toxicities compared to monotherapy, the most common of which are nonspecific rashes, pruritus, and vitiligo [5]. The toxicities appear earlier compared to anti-PD-1 monotherapy. In contrast to their frequent occurrence, the severity is usually low [6]. Data from the trial RELATIVITY-047 show that cutaneous toxicity in patients receiving the recently approved combination of relatlimab/nivolumab seems to be slightly higher compared with nivolumab monotherapy. However, severe grade III and IV toxicities were similar in the two groups. Nonetheless, further studies are needed to evaluate the incidence of irAE in patients receiving this novel treatment combination [7].

**Figure 1:**
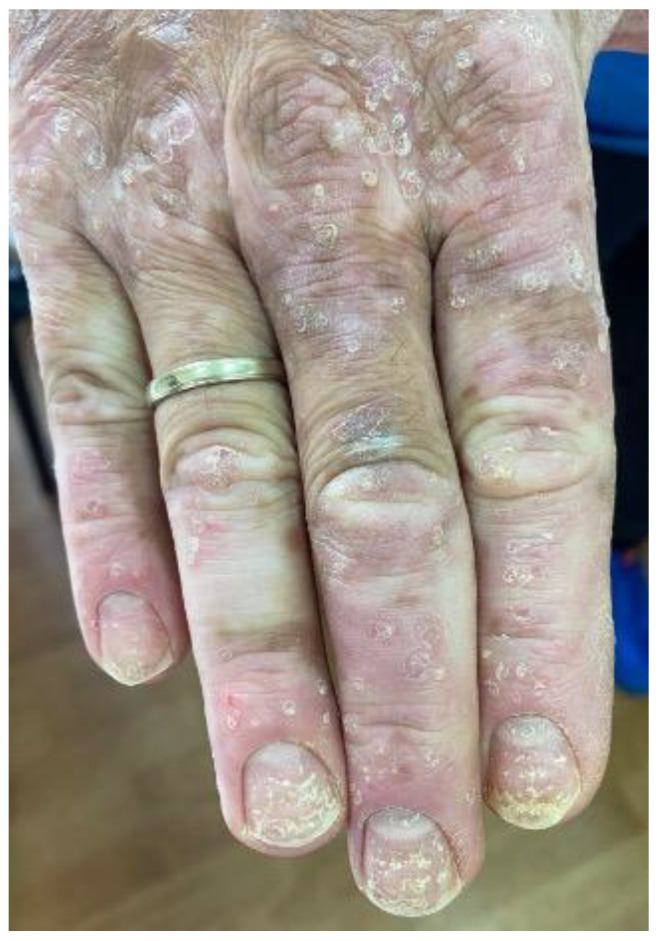
A melanoma patient developing plaque psoriasis, nail psoriasis and vitiligo after ICB treatment.

Notably, the vast majority of cutaneous irAEs have been described in patients with melanoma. A recent study of 8637 patients under ICBs and 8637 matched controls showed that patients with melanoma and renal cell carcinoma were at higher risk of cutaneous irAEs compared to patients with other solid malignancies [8]. This emphasizes the need for close dermatologic follow-ups in this group of patients. Moreover, a significant proportion of melanoma patients exhibited more than one cutaneous toxicity (Figure 1). Hwang et al., in a retrospective study of 82 metastatic melanoma patients, reported that lichenoid reactions, eczema, and vitiligo tend to appear together in patients under anti-PD-1 therapy, suggesting a common pathogenetic mechanism mediated by lymphocyte damage [9]. In a more recent study of 762 cases, the European Academy of Dermatology and Venereology (EADV) task force group for cancer patients demonstrated that melanoma patients developed most commonly multiple skin toxicities, suggesting an increased awareness in this group of patients [10].

The factors that can predict the development of cutaneous toxicities are largely unknown. It has been reported that patients with preexisting autoimmune diseases are at an increased risk of experiencing flare-ups following ICB administration [11]. Similarly, personal and familial histories of psoriasis are significant predictors of psoriasis development following ICB treatment [12].

## 3. Systemic Agents Used in the Treatment of Cutaneous Immune-Related Adverse Events

Most cutaneous irAEs are reversible with the systematic use of corticosteroids. However, glucocorticosteroids are associated with potential multiple side effects and may have an impact on anti-tumor response. It has been reported that steroids before or early during ICB treatment may negatively affect outcomes [13,14]. A recent meta-analysis including 4045 patients treated with ICB showed that patients taking steroids were at increased risk of death and progression compared to those not taking steroids [15]. However, the main negative effect on overall survival (OS) was associated with patients taking steroids for supportive care or brain metastases, whereas steroids used to mitigate adverse events did not negatively affect OS. Nevertheless, steroid-sparing agents should be preferred, whereas tapering glucocorticoids to the lowest effective dose within weeks or as soon as improvement is achieved is desirable. Table 1 summarizes systemic agents used for the treatment of cutaneous ICB-induced toxicities.

## 4. Cutaneous Immune-Related Adverse Events

### 4.1. Morbilliform (Maculopapular) Rash

#### 4.1.1. Incidence

Cutaneous toxicities are often described as a “rash” in oncology reports on irAEs, which makes estimating their true prevalence difficult. It has been reported that morbilliform rashes are very common in patients treated with anti-CTLA4 therapy, affecting up to 14 to 26 percent of patients receiving ipilimumab and up to 55 percent of patients receiving combination anti-CTLA4/PD-1 inhibition therapy [36]. The frequency rates are lower for anti-PD-1 and anti-PD-L1 therapies, affecting up to 20 percent of patients [37]. Grade-3 reactions are rare, occurring in less than two percent of patients of ICB monotherapy and less than five percent of patients receiving combination regimens [37].

A recent large study from the EADV Task Force of Dermatology for Cancer Patients showed that morbilliform eruption is the most frequent cutaneous irAE in melanoma patients, with 32.3 percent of patients affected [10]. Given that anti-CTLA-4 agents are used to treat melanoma patients, it is expected that these reactions will mainly affect melanoma cases that are routinely exposed to these agents in combination with PD-1 blocking agents. However, the study showed a significant association between macular rashes and melanoma over non-small cell lung cancer, which was used as a reference category in multivariate models. This suggests that melanoma pathogenesis and macular rash development share common immunologic pathways to which ICB treatments could contribute to further deterioration.

#### 4.1.2. Clinicopathological Characteristics

**Figure 2:**
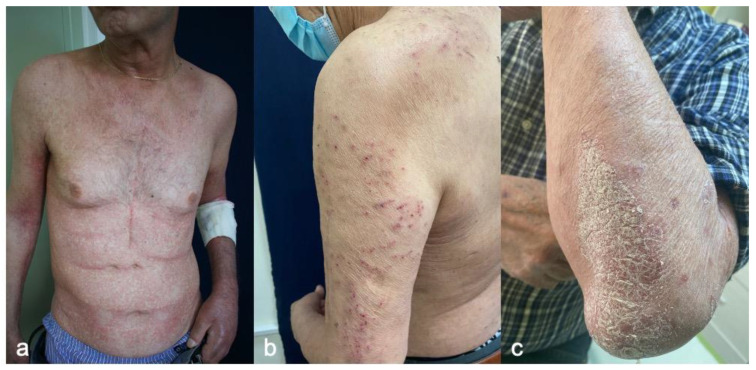
(**a**). Melanoma patient with ICB-induced macular (morbilliform) rash. (**b**). Multiple excoriations secondary to severe pruritus in a melanoma patient treated with ICB. (**c**). ICB-induced plaque psoriasis.

A morbilliform rash is one of the first cutaneous eruptions to appear. Phillips GS et al., in a large retrospective study of 285 patients with cutaneous iRAEs, showed that macular rashes presented earlier than other cutaneous toxicities (62 days vs. 133 days, *p <* 0.001) [21]. Patients present erythematous macules and papules congregating into plaques accompanied by pruritus in most cases (Figure 2a). The lesions initially appear on the trunk, then later extend to the extensor surfaces of the extremities. The face, scalp, palms, and soles are generally spared. Physicians need to be aware that morbilliform rash may represent the initial manifestation of severe skin disorders, such as Stevens–Johnson Syndrome (SJS) or Bullous Pemphigoid (BP) [38]. Histopathologic examinations reveal a superficial, perivascular lymphocytic infiltrate of CD4+ T cells with interstitial eosinophils, with or without epidermal spongiosis and papillary dermal edema [39]. In melanoma patients treated with an anti-CTLA-4 antibody, peripheral eosinophils were observed at the time of skin eruption [40].

#### 4.1.3. Management

The management of skin eruptions adjusts to the severity of the rash and is based on Common Terminology Criteria for Adverse Events (CTCAE): grade 1 (<10% body surface area affected [BSA], mild), grade 2 (10–30% BSA, moderate) grade 3 (>30% BSA, severe), and grade 4 (life-threatening eruption, urgent intervention needed) [16]. Morbilliform eruptions are typically mild and self-limiting. Skin biopsy is not mandatory and must be reserved only for atypical rashes. Symptomatic treatment with topical steroids on the affected areas and moisturizers with or without oral antihistamines is recommended. Grade-1 and -2 reactions do not warrant treatment discontinuation. For severe grade-3 reactions, interrupting the treatment until the rash has reduced to grade-1 must be considered. Oral steroids (0.5–1 mg/kg/day of prednisone equivalent) tapered over four weeks are recommended in addition to topical treatments. The re-administration of ICB is usually tolerated when the rash has reduced to grade-1, and the dose of prednisolone is less or equal to 10 mg [41].

### 4.2. Pruritus

#### 4.2.1. Incidence

According to a recent meta-analysis, pruritus was found to be one of the most common irAEs, affecting 26 percent of patients, with one percent experiencing severe grade-3 symptoms [42]. Pruritus is most frequent in patients receiving anti-CTLA4 or combination regiments, affecting 25–36 percent and 33–47 percent of patients, respectively. An increased incidence of pruritus has also been reported for the recently approved combination of LAG-3/PD-1 for melanoma compared to anti-PD-1 monotherapy (23.4% vs. 15.9% for Relatlimab-Nivolumab and Nivolumab respectively) [7]. Pruritus can develop at any time during treatment. One study observed a median of three treatment cycles before pruritus appeared, with a broad range of one to 17 cycles [36].

#### 4.2.2. Clinicopathologic Characteristics

Pruritus usually accompanies other skin toxicities, such as macular rash and eczematous or lichenoid reactions. However, it can also be associated with normal-appearing skin. In such cases, secondary changes such as erosions, ulcerations, prurigo nodules, or skin superinfection may be detected (Figure 2b). Most patients present a low-grade rash. Pruritus can significantly impact patient quality of life and instrumental activities of daily living.

#### 4.2.3. Treatment

Mild pruritus should be treated with topical solutions, such as topical moisturizers and medium-to-high potency steroids, as well as topical camphor/menthol for symptomatic relief. Non-sedating antihistamines can be added to the regimen. In the event of refractory to antihistamines, pruritus GAPA agonists such as gabapentin or pregabalin can be given, followed by low doses of systemic steroids (10 mg of prednisolone or equivalent). UVB-NB phototherapy has also been successfully used to treat ICB-induced pruritus [43]. In addition, anecdotal reports suggest that other medications with anti-pruritic effects, such as aprepitant, omalizumab, and dupilumab, may also be effective (Table 1) [44,45]. Basic laboratory evaluations, such as complete blood count and renal and liver function assessments, should be considered in refractory cases in addition to skin biopsy and direct immunofluorescence to rule out the early pre-bullous stages of BP.

### 4.3. Psoriasiform Reactions

#### 4.3.1. Incidence

The development or exacerbation of psoriasis is a well-established side effect of anti-PD-1/PD-L1 therapies, but it is rarely reported during treatment with ipilimumab [46]. The actual incidence of ICB-induced psoriasis still needs to be determined. Psoriatic arthritis can also occur in 8.1 percent of patients who develop psoriasis during ICB therapy. Psoriasis appears after a median of 5–12 weeks after the initiation of treatment. The exacerbation of preexisting disease occurs earlier and after fewer total infusions compared to de novo psoriasis [47].

#### 4.3.2. Clinicopathologic Characteristics

Patients most commonly present similarly to idiopathic psoriasis with typical erythematous plaques, namely with silvery scales on the elbows and knees (Figure 2c). However, all types of psoriasis have been reported, including pustular, erythrodermic, inverse, and nail psoriasis [47]. Patients treated with ICB often develop more than one clinical subtype of psoriasis [47].

Histologically, psoriasiform epidermal hyperplasia and Munro microabscesses tend to present. Features of spongiosis and eosinophils are described in 40 percent of cases. Psoriasis can have an overlapping histopathologic and immune profile with some forms of atopic spongiotic dermatitis [48]. It has been reported that the PD-1 blockade causes a shift to a pro-inflammatory Th-1/Th-17 response, increasing levels of tumor necrosis factor-alpha (TNF-alpha), interleukins 2, 6, 17 and interferon-γ [49].

#### 4.3.3. Treatment

Topical treatments, including steroids, salicylic acid, and vitamin-D analogs, are routinely used for grade-1 and grade-2 reactions. UVB-NB phototherapy can be considered in selected patients with melanoma with recalcitrant lesions or grade-3 disease and only after close monitoring of skin lesions. Alternatively, systematic treatment can be used on a case-by-case basis. Acitretin is a safe option for cancer patients and can be used as a first-line treatment. Low doses of methotrexate (10–15 mg/week) can be considered, with the exception of non-melanoma skin cancers [17]. Long-term safety data are lacking for novel agents such as apremilast, anti-TNFa, anti-IL17, and anti-IL23. Small case series have been reported and should be considered on a case-by-case basis. TNF-a inhibitors are commonly used to treat ICB-associated colitis, and it has been reported that TNF-a inhibition may improve the safety profile of checkpoint inhibitor therapy without decreasing its efficacy (Table 1). However, further studies on ICB-psoriasis are required to confirm these findings [50].

### 4.4. Lichen Planus-like Rash

#### 4.4.1. Incidence

The overall incidence of lichen planus-like reactions is less than 17 percent of all cutaneous irAEs and is mainly observed in the presence of anti-PD-1/PD-L1 monoclonal antibodies [30]. Disease onset can vary, ranging from three days up to a year after treatment initiation [30].

#### 4.4.2. Clinicopathologic Characteristics

**Figure 3:**
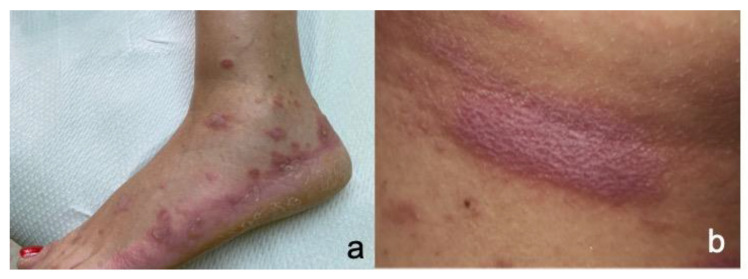
(**a**) Violaceous papules and plaques typical of lichen planus on the acral sites of a melanoma patient treated with ICB. (**b**) Wickham striae on a lichen planus plaque.

Patients present lichen planus-like eruptions consisting of flat-topped, shiny, violaceous papules or plaques mainly located on the extremities and the trunk (Figure 3a). The plaques can be crossed by white reticulated lines called Wickham striae (Figure 3b). The rash can be accompanied by severe pruritus.

Mucous membranes, such as the oral and genital mucosa, can also be affected, either in conjunction with skin lesions or independently. Wickham’s striae represent the most common presentation on the oral mucosa, followed by erosive mucosal lesions. Atrophic white plaques on the genitals, typical of lichen atrophicus, can appear on the genitals. Nail involvement with dystrophy, ridging, and hyperkeratosis have also been reported. Several other clinical types of lichen planus have been described in case reports and case series, including erosive and hypertrophic variants, bullous lichen planus pemphigoids, and inverse lichen planus [51].

Pathologic evaluations demonstrate the typical features of classic lichen planus. These include lichenoid and interface lymphocytic infiltrates, basal vacuolar changes, and hyperkeratosis. Unlike typical lichen planus, apoptotic keratinocytes—associated with variable parakeratosis, necrosis, epidermal spongiosis, and eosinophils—may be seen. Recent reports also describe a clinical type of hypertrophic lichen planus with squamous cell carcinoma-like histology [51].

#### 4.4.3. Treatment

Immunotherapy can be maintained in most cases. Potent or superpotent topical steroids are used for grade-1 and 2 reactions. In patients with grade-3 disease, systemic steroids (0.5–1 mg kg/day) are recommended. Retinoids (acitretin 10–30 mg/day), methotrexate, and azathioprine treatments can be considered in refractory cases based on case series and reports (Table 1) [52]. UVB-NB can also be used with the precautions already discussed for melanoma patients.

### 4.5. Eczematous Reactions

#### 4.5.1. Incidence

Eczematous dermatitis is a common reaction to anti-PD-1 inhibitors, affecting approximately 17 percent of patients [53]. However, its exact incidence cannot be evaluated accurately because non-specialists commonly misdiagnose these reactions as morbilliform eruptions.

#### 4.5.2. Clinicopathologic Characteristics

Patients present erythematous papule patches and/or plaques accompanied by pruritus. The lesions are mainly located on the trunk and the extremities. Secondary excoriations may be prominent. Severe cases of prurigo nodularis have also been reported [54]. Eczematous eruptions may be the first sign of bullous pemphigoids, and in refractory cases, a skin biopsy and direct immunofluorescence are advised [55]. Histologically, ICB-induced eczema exhibits epidermal spongiosis, papillary dermal edema, and a perivascular lymphocytic infiltrate with an increased number of eosinophils.

#### 4.5.3. Therapy

Similar to the treatment algorithm for lichen planus-like eruptions, tropical potent or superpotent steroids are the mainstay of treatments for grade-1 and grade-2 diseases. Patients should be advised to use emollients daily. Pruritus can be treated with antihistamines. In the event of a severe reaction, the addition of low-dose prednisolone should be considered.

### 4.6. Bullous Pemphigoid (BP)

#### 4.6.1. Incidence

The overall incidence of BP is between one and five percent. It is typically associated with anti-PD-1/PD-L1 agents. Anti-CTLA-4 antibodies are only rarely implicated in the development of BP or other bullous disorders. Time to disease onset can vary, but most data indicates that it has an overall greater latency of onset than other skin irAEs [56].

#### 4.6.2. Clinicopathologic Characteristics

**Figure 4:**
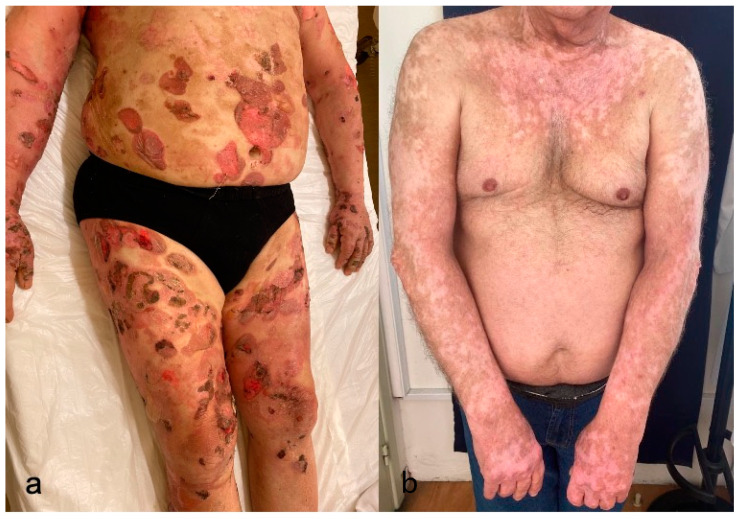
(**a**). ICB-induced bullous pemphigoid (**b**). Vitiligo distributed on sun-exposed areas.

The clinical characteristics of ICB-induced BP strongly resemble those of classic BP. A pro-bullous phase with pruritus, eczematous reactions, urticarial lesions, or nonspecific macular rash may precede the presence of tense bullae. The rash mainly affects the trunk and extremities (Figure 4a). Mucosal involvement is uncommon, but it is more common than with classic BP [57,58]. Other types of autoimmune bullous diseases, such as pemphigus, linear IgA bullous dermatosis, and dermatitis herpetiform, have been seldom reported in the setting of immunotherapy.

The histology of BP is characterized by a subepidermal split and numerous eosinophils. The dermis is infiltrated by lymphocytes, eosinophils, and neutrophils. Direct immunofluorescence reveals the linear deposition of IgG and C3 at the dermo-epidermal function, whereas salt-split skin analysis shows the deposition of anti-immunoglobulin G antibodies on the epidermal side of the bulla. Enzyme-linked immunosorbent assays reveal antibodies against the hemidesmosomal proteins (BP180 and BP230) [59].

#### 4.6.3. Treatment

BP may persist for several months despite treatment discontinuation. Therapeutic approaches should be made on an individualized basis. Patients must be closely monitored. For grade-1 reactions, ICB can be continued, and superpotent topical steroids can be applied. If the reaction worsens to grade 2, oral steroids should be administered (prednisolone 0.5 mg/kd/day), and ICB should be withheld until the rash returns to grade 1. Hospitalization should be considered for severe grade 3 and grade 4 reactions. Higher doses of prednisolone should be administered, and the addition of a steroid-sparing agent can be considered. Doxycycline with or without niacinamide can be used in mild cases, while methotrexate, dapsone, and plasmapheresis, as well as monoclonal antibodies such as omalizumab or Rituximab, are preferred for recalcitrant cases (Table 1). Patients can be rechallenged with ICB under a low dose of prednisolone when the reaction has returned to grade 1.

### 4.7. Vitiligo

#### 4.7.1. Incidence

Vitiligo is primarily identified in patients with melanoma. However, it has been reported in relation to other malignancies, including renal cell carcinoma and NSCLC [60,61]. A systematic review and meta-analysis reported the overall incidence in melanoma patients to be only two percent of melanoma patients treated with immunotherapy [62]. However, recent data indicate a higher incidence of two to nine percent of patients treated with anti-CTLA4 and up to 24 percent of those treated with anti-PD-1 monotherapy or combination treatments [38]. Depigmentation typically develops seven to 65 weeks after the initiation of therapy.

#### 4.7.2. Clinicopathologic Characteristics

A wide clinical spectrum of vitiligo has been reported in melanoma case series. Typical hypopigmented patches with symmetrical distribution are the most prominent clinical presentation. Depigmentation of the eyelashes, eyebrows, or hair has also been reported. In addition to the classic form of vitiligo, patterns of small freckle-like lesions on chronically sun-exposed areas and the development of halo nevi have also been reported (Figure 4b) [63].

ICB-induced vitiligo has been linked to a cross-reaction against antigens shared by normal melanocytes and melanoma cells, such as tyrosinase, MART-1, and GP100, as well as tyrosinase-related proteins 1 and 2 or tyrosinase. The histology reveals an absence of epidermal melanocytes at the dermo-epidermal junction and a CD8 T-cell infiltrate with overexpressed CXCR3 in addition to raised interferon-γ and tumor necrosis factor levels [64].

#### 4.7.3. Treatment

Treatment discontinuation is not required, even in the event of extensive disease. However, patients must be warned before starting ICB that this toxicity, although not life-threatening, is rarely reversible. Topical steroids and/or tacrolimus pimecrolimus creams can be prescribed for localized disease. Repigmentation after the discontinuation of treatment has been reported and may be associated with disease progression or recurrence [65].

### 4.8. Sarcoidosis

#### 4.8.1. Incidence

Although it is a rare cutaneous irAE, sarcoidosis has been observed to present more frequently in melanoma patients treated with ICB than in those treated for other cancers [66,67]. Disease onset occurs at least one month after treatment initiation. As with other skin reactions, timing can vary from zero to 24 months [68].

#### 4.8.2. Clinicopathologic Characteristics

Solitary or multiple violaceous to brown papules, plaques, or subcutaneous nodules are the typical clinical characteristics of sarcoidal lesions. The rash can appear on the trunk or the extremities, as well as in the regions of prior scars or tattoos. Panniculitis has been described as ‘Lofgren syndrome’ with coincident polyarthralgia [69]. A diagnosis of a sarcoid-like reaction should prompt a workup for pulmonary disease, as this population is often asymptomatic. Pulmonary involvement can be clinically difficult to differentiate from the disease progression of melanoma.

The pathology reveals nodular collections of epithelioid histocytes with scant accompanying lymphocytes, such as sarcoidal granulomas in the dermis.

#### 4.8.3. Treatment

Cutaneous lesions can be treated with topical or intralesional steroids or synthetic anti-malarial drugs.

### 4.9. Severe Cutaneous Adverse Reactions

#### 4.9.1. Incidence

Severe cutaneous adverse reactions (SCARs) are events that often require hospitalization and can be fatal. These include Stevens-Johnson syndrome (SJ), toxic epidermal necrolysis (TEN), drug rash with eosinophilia and systemic syndrome (DRESS), and acute generalized exanthematous pustulosis (AGEP). Overall, ICB-induced SCARs seem to be infrequent. The majority of SJS/TEN cases occur early, with onset during the first or second treatment cycle [70]. However, unlike classic SJS/TEN, ICB-induced reactions can develop months after starting ICBs [71].

#### 4.9.2. Clinicopathologic Characteristics

Patients may develop a morbilliform eruption or confluent erythema followed by the development of targetoid patches, flaccid bullae with a positive Nikolsky sign, and mucous membrane ulcerations similar to classical SJS/TEN. Cases of DRESS are associated with facial swelling, diffuse erythema, fever, lymphadenopathy, eosinophilia and internal organ involvement (hepatitis, pneumonitis, nephritis, colitis, myocarditis, pericarditis). The occurrence of AGEP has been observed with anti-CTLA-4 or anti-PD-1 therapies and is characterized by the sudden development of numerous small pustules overlying erythematous and edematous plaques. AGEP must be differentiated from pustular psoriasis and acneiform drug eruptions [72].

The pathology of SJS/TEN cases reveals epidermal necrosis and keratinocyte apoptosis associated with vacuolar interface alteration, subepidermal lymphocytes, and cleavage along the dermal-epidermal plane. CD8-positive T-cells are present within the dermal-epidermal junction, as are increases in the PD-L1 expression of lymphocytes and keratinocytes in the epidermis [55].

#### 4.9.3. Treatment

Reports note a high prevalence of death in young adults with SCARs, especially TEN (83% in adults aged 40–49) [71]. Most cases require the permanent discontinuation of ICB and immediate hospitalization. Supportive care is vital, including careful fluid and electrolyte maintenance, nutritional support, wound care, and ophthalmology consultations. Intravenous systemic steroids are recommended. Cyclosporine or intravenous immunoglobulin have also been used in non-responders. Some anecdotal evidence exists for the use of TNF-a inhibitors in treating SJS/TEN-like reactions to immunotherapy.

### 4.10. Other Reactions

There are several skin reactions related to ICBs. A skin biopsy is mandatory to diagnose clinically atypical reactions. Table 2 presents the most significant cutaneous toxicities associated with ICBs in case reports or case series.

## 5. Survival Related to Cutaneous Immune-Related Adverse Events

A growing body of research has examined the association between cutaneous toxicities and disease survival in recent years. A retrospective study of 7008 cancer patients who developed ICB-induced cutaneous toxicity showed that the development of pruritus, drug eruption, xerosis, nonspecific rashes, and any cirAE as a group were all significantly protective against mortality. This suggests that these reactions are strongly associated with the response to ICB therapy and patient survival [73]. However, the prognostic significance of specific cutaneous reactions and their associations with different malignancies remains unclear.

The association of vitiligo with a melanoma diagnosis or melanoma regression is well known and believed to be a consequence of an immune response against antigens shared by melanoma and normal melanocytes [74]. Several studies have linked leukoderma to regressing melanoma, indicating that patients with skin depigmentation should be closely examined for the presence of primary tumors [75,76]. Vitiligo has also been significantly associated with a favorable prognosis with the use of ICB therapy in melanoma patients [77]. Vitiligo repigmentation, developing in association with tumor relapse, has also been reported among nivolumab-treated melanoma patients [78].

Maculopapular eruptions have been associated with improved overall survival in patients treated with nivolumab and pembrolizumab, with a statistically significant improvement across all grades of rash severity [79]. Thomson et al. reported that specific cutaneous irAEs, such as lichenoid or psoriasiform morphologies, may hold prognostic significance, whereas others, such as papular eruptions and isolated pruritus, were not meaningfully associated with survival outcomes [80]. Nikolaou et al. reported that the presence of guttate psoriasis and psoriasis affecting over 10 percent of BSA were both associated with better response rates to immunotherapy compared to other types of psoriasis and mild symptomatology, respectively. It has also been reported that BP and sarcoidosis may be associated with better clinical outcomes in some patients with metastatic melanoma [69,81]. Further, lichenoid dermatitis has been associated with improved therapeutic responses, longer progression-free survival, and longer overall survival times compared to patients who did not develop lichenoid dermatitis [82].

Other reports indicate that patients with improved progression-free survival experience more than one form of cutaneous irAE (i.e., eczema, lichenoid reaction, or vitiligo) [83]. Finally, the positive effects of multiple toxicities were reported in a melanoma cohort, where 69 percent of patients with one cutaneous toxicity were alive at the end of follow-up compared to 81 percent of patients who developed multiple cutaneous irAEs [84]. Nevertheless, it is important to note that while many retrospective analyses support the association between cutaneous toxicities and patient survival, these findings should still be viewed with caution since some of these positive correlations may be due to bias.

## 6. Conclusions

Skin toxicities represent the most common class of toxicities in patients under ICBs. They are characterized by a polymorphous clinical spectrum. However, our understanding of the pathophysiology of these reactions remains incomplete, despite rational, mechanistic hypotheses. Compared to skin specialists, physicians not experienced in treating skin diseases often overestimate the severity of skin rashes and are more prone to hold or even permanently discontinue treatment due to skin toxicities [85]. At the same time, the existing grading system based on the affected BSA is not always clinically relevant to the severity of the disease. SJS affecting 10 percent of the BSA is a clinical emergency, whereas a macular rash affecting 30 percent of the BSA can be managed without significant interventions. Likewise, psoriasis affecting 20 percent of the BSA can be considered milder than palmar psoriasis affecting two percent of the BSA. This can significantly impact a patient’s access to potentially life-saving treatments. Therefore, the role of dermatologists in oncological multidisciplinary care is very important to achieving treatment coherence and improving patient quality of life. The overall goal is to promptly recognize and manage these toxicities, thus enabling patients to remain on their potentially life-saving cancer therapies.

## Figures and Tables

**Table 1:** Most commonly used systemic agents used for the treatment of cutaneous irAEs.

Systemic Treatment	Indications	Dose	Comments
Glucocorticosteroids [11,12,13,16]	Maculopapular rash Lichen Planus like rash Eczematous reactions Pruritus Bullous dermatoses SCARs (SJS, TEN, DRESS) Other toxicities (neutrophilic dermatoses, vasculitis, lupus erythematosus, dermatomyositis)	Grade 2: prednisone (or equivalent) 0.5–1 mg/kg tapered over at least 4 weeks Grade 3: (prednisone 1 to 2 mg/kg/d or methylprednisolone 1 to 2 mg/kg/d).	- Steroids before or early during ICI treatment may negatively affect outcomes [11,12] - Low doses of glucocorticoids used to treat. irAEs do not affect response rates [13] - Steroid-sparing agents should be preferred - Tapering glucocorticoids to the lowest effective dose within weeks or as soon as improvement is achieved is desirable
Methotrexate [17]	Psoriasiform Dermatomyositis Bullous Pemphigoid	10–25 mg oral or subcutaneous once weekly	- Carries no increased risk for cancer recurrence with the slight exception of non-melanoma skin cancers
Acitretin	Psoriasiform Lichenoid	25–30 mg oral daily	- First line option. There is no evidence of an increased risk of solid cancers.
Anti-TNF [18,19,20]	SJS/TEN (Infliximab) Maculopapular Lichenoid (Refractory Grade3, infliximab) Psoriasis	Infliximab 5 mg/kg infusion Adalimumab	- Inhibition of TNFα synergizes with checkpoint blockade, leading to enhanced CD8 T cell immunity in animal models. - Short-term TNF inhibition should not compromise ICB efficacy [21]. - A correlation between the use of TNFα blockers and decreased cancer survival after ICI has been reported
Anti-IL6 [22]	Maculopapular rash Lichenoid rash Generalized morphea	Tocilizumab 162 mg subcutaneous injection every 2 weeks	- For corticosteroid resistant maculopapular or lichenoid rash - Check serum levels of IL-6 to assess for eligibility
Dupilumab [21,23,24]	Bullous pemphigoid Eczematous dermatitis	600 mg loading dose and 300 mg subcutaneous injection administered every other week thereafter	- Dupilumab treatment carries lower risks of systemic immunosuppression - The adverse effects associated with dupilumab are relatively mild
Omalizumab [25]	Bullous pemphigoid Eczematous dermatitis Urticaria Pruritus	300-mg monthly injections	- Immunoglobulin E (IgE) blocker - It is well tolerated in cancer patients with pruritus related to ICBs
Rituximab [26,27]	Bullous pemphigoid	375 mg/m^2^ once weekly for 4 weeks	- Anti-CD20 antibodies had no effect on tumor growth, response to PD-1 inhibition or survival in murine models. - Case reports support the role of Rituximab in the treatment of ICI-induced BP
IVIG [28,29]	Dermatomyositis Bullous pemphigoid SJS/TEN	BP: IVIG 1–2 g/kg every 4 weeks (along with steroids) Dermatomyositis: 0.4 mg/kg daily for 5 days monthly SJS/TEN: 1–1.5 g/kg single infusion	- Case reports - Safe to administer in a setting of malignancy
Apremilast [21,30]	Psoriasiform	30 mg twice daily	- Mostly interacts with the innate immune system and is considered a relatively safe option for cancer patients. Long-term safety data are lacking.
Anti-IL23 [30]	Psoriasiform	Guselkumab: 100 mg subcutaneous injection at weeks 0 and 4 and then every 8 weeks. Risankizumab: 150 mg subcutaneous injection at weeks 0 and 4 and then every 12 weeks Tildrakizumab: 100 mg subcutaneous injection at weeks 0, 4 and every 12 weeks thereafter.	- Myeloid cell production of IL-12 was shown to be essential for anti-PD-1 therapeutic efficacy in mouse models. - In mouse models, IL-23 deficiency provided tumor protection and improved survival compared to WT mice. - High tumor-derived IL23A gene expressions have been correlated with poor survival in osteosarcoma patients. - Anti-IL-23 may be considered in severe or refractory cases - Minimal immunosuppressive effect - Case reports with risankizumab and guselkumab have been published [31,32]
Anti-IL12/23 [33]	Psoriasiform	Ustekinumab: 45 mg subcutaneous injection at weeks 0 and 4 and then every 12 weeks	- Interfering with IL-12 might restrict the antitumor immune response to CPI treatment. Myeloid cell production of IL-12 was shown to be essential for anti-PD-1 therapeutic efficacy in mouse models.
Anti-IL17 [34,35]	Psoriasiform	Ixekizumab: 160 mg by subcutaneous at week 0, followed by 80 mg at weeks 2, 4, 6, 8, 10, and 12, then every 4 weeks. Secukinumab: 300 mg subcutaneous injection at weeks 0, 1, 2, 3 and 4 and then monthly. Brodalumab: 210 mg subcutaneous injection at weeks 0, 1, and 2 and then every 2 weeks. Bimekizumab: 320 mg subcutaneous injection at weeks 0, 4, 8, 12, 16 and every 8 weeks thereafter.	- Should be generally avoided due to the known GI side effects. - In preclinical mouse models, IL-17 can initiate both pro- and antitumor immune effects - Secukinumab has shown profound therapeutic efficacy in the treatment of cancer patients developing ICB-induced psoriasis - May be considered on a case-by-case basis and multidisciplinary approach for refractory psoriasis - Case reports with ixekizumab and secukinumab have been published

**Table 2:** Uncommon cutaneous irAEs.

Cutaneous Toxicity	Clinical Presentation	Treatment
Alopecia areata	Non-scarring round patches of alopecia Alopecia totalis: Entire scalp and eyebrow involvement Alopecia universalis: Loss of total body hair.	- Topical or intralesional corticosteroids
Scleroderma	Skin tightening with thickening. Digital swelling. Xerosis. Periungual erythema. Fatigue. Muscle atrophy/weakness. Raynaud phenomenon	- Systemic corticosteroids - Hydroxychloroquine - Immunosuppressants (e.g., Mycophenolate mofetil) - IVIG
Leukocytoclastic vasculitis	Palpable purpura on the extremities	- -Systemic corticosteroids - -Monitor for systemic involvement - -Hydroxychloroquine - -Methotrexate
Acne-like lesions	Follicular papules and pustules located on the efface and trunk Rosacea like reactions	- Doxycycline or minocycline 100–200 mg/day
Neutrophilic Dermatoses *Sweet’s syndromePyoderma gagrenosum*	Violaceous, edematous tender papules and plaques Head and neck, extremities Fever, malaise, arthralgia Pustules that progress to ulcers with violaceous and undermined borders, often painful May occur at sites of trauma	- Systemic corticosteroids - Anti-neutrophilic agents (dapsone and colchicine)
SICA syndrome	Dry mouth, dry eyes	- Systemic corticosteroids - Saliva substitutes, Artificial tears
Grover Disease (Transient acantholytic dermatosis)	Pruritic erythematous papules, keratotic papules or papilovesicular eruption distributed on the central chest and back	- High potency topical steroids bid for 2 weeks - Antihistamines - Topical vitamin D analogs bid for 4 weeks - Systemic corticosteroids (e.g., prednisone 20–40 mg qd) - Systemic retinoids (isotretinoin 10–40 mg qd)
Ichthyosis	Symmetric fish-like scaling, platelike brow scales on the limps, rough, dry skin on the trunk. Skin folds are spared	- Topical emollients - Acitretin 10–25 mg/day

## Data Availability

The data can be shared up on request.
